# Importin-13 genetic variation is associated with improved airway responsiveness in childhood asthma

**DOI:** 10.1186/1465-9921-10-67

**Published:** 2009-07-20

**Authors:** Benjamin A Raby, Kristel Van Steen, Jessica Lasky-Su, Kelan Tantisira, Feige Kaplan, Scott T Weiss

**Affiliations:** 1 Channing Laboratory, Department of Medicine, Brigham and Women's Hospital, Boston, Massachusetts, USA; 2 Division of Pulmonary and Critical Care Medicine, Brigham and Women's Hospital, Boston, Massachusetts, USA; 3 Harvard Medical School, Boston, Massachusetts, USA; 4 Center for Genomic Medicine, Brigham and Women's Hospital, Boston Massachusetts, USA; 5 Department of Oto-rhino-laryngology & Department of Applied Mathematics and Computer Science, University of Ghent, Belgium; 6 Departments of Human Genetics and Pediatrics, McGill University, Montreal Quebec, Canada

## Abstract

**Background:**

Glucocorticoid function is dependent on efficient translocation of the glucocorticoid receptor (GR) from the cytoplasm to the nucleus of cells. Importin-13 (IPO13) is a nuclear transport receptor that mediates nuclear entry of GR. In airway epithelial cells, inhibition of IPO13 expression prevents nuclear entry of GR and abrogates anti-inflammatory effects of glucocorticoids. Impaired nuclear entry of GR has been documented in steroid-non-responsive asthmatics. We hypothesize that common IPO13 genetic variation influences the anti-inflammatory effects of inhaled corticosteroids for the treatment of asthma, as measured by change in methacholine airway hyperresponsiveness (AHR-PC_20_).

**Methods:**

10 polymorphisms were evaluated in 654 children with mild-to-moderate asthma participating in the Childhood Asthma Management Program (CAMP), a clinical trial of inhaled anti-inflammatory medications (budesonide and nedocromil). Population-based association tests with repeated measures of PC_20 _were performed using mixed models and confirmed using family-based tests of association.

**Results:**

Among participants randomized to placebo or nedocromil, IPO13 polymorphisms were associated with improved PC_20 _(i.e. less AHR), with subjects harboring minor alleles demonstrating an average 1.51–2.17 fold increase in mean PC_20 _at 8-months post-randomization that persisted over four years of observation (p = 0.01–0.005). This improvement was similar to that among children treated with long-term inhaled corticosteroids. There was no additional improvement in PC_20 _by IPO13 variants among children treated with inhaled corticosteroids.

**Conclusion:**

IPO13 variation is associated with improved AHR in asthmatic children. The degree of this improvement is similar to that observed with long-term inhaled corticosteroid treatment, suggesting that IPO13 variation may improve nuclear bioavailability of endogenous glucocorticoids.

## Background

Endogenous glucocorticoids (GCs) serve a broad range of biological and physiological processes, including metabolic control, induction of anti-inflammatory cascades, and fetal organ maturation. In the context of the lung, GCs modulate airway branching morphogenesis[1,2], augment production of surfactant in late gestation[3], and promote alveolar formation[4]. As a result of their potent anti-inflammatory properties, exogenous GCs also serve as the most commonly used treatment for the long-term control of asthma[5] by effectively reducing airway hyperresponsiveness (AHR) and asthma symptoms[6], preventing exacerbations[7], and reducing asthma-associated mortality[8].

Though the precise molecular mechanisms that explain the diverse effects of GC have yet to be completely defined, nearly all GC effects result through cell-specific transcriptional regulation following binding of GC (in complex with the glucocorticoid receptor – GR) to positive and negative GC response elements in the promoter region of genes[9,10]. In its unbound state, GC is typically sequestered in the cytoplasm by molecular chaperones[9]. To access its genomic targets, GC-GR complexes must first pass from the cytoplasm to the nucleus through nuclear pore complexes. This process of GC- receptor shuttling across the nuclear-cytoplasmic membrane is tightly regulated by the interaction of cell-specific nuclear transport factors with cognate nuclear localization sequences[11]. Importin-13 (IPO13) was initially cloned in a search for GC-regulated genes important in lung development[12], and found to be differentially expressed during fetal lung growth with enrichment in lung epithelium relative to the mesenchyme. More recently, we (FK) demonstrated that IPO13 silencing prevents GC transport across the cytoplasmic-nuclear membrane in airway epithelium and abrogates GC-induced anti-inflammatory responses, suggesting that IPO13 is a critical nuclear transporter of GC receptor in the airway epithelium[13].

Although most asthmatics demonstrate an improvement in asthma control with long-term inhaled GC therapy, large inter-individual variation in response to inhaled GCs is well documented [14-16]. While some patients do respond to higher doses than normally prescribed, the administration of these doses for prolonged periods can have marked adverse effects. The identification and characterization of genetic determinants of GC responsiveness would provide insight into GC pharmacology and asthma pathogenesis. Given its demonstrated ability to mediate anti-inflammatory GC effects (particularly in airway epithelium), we considered that IPO13 represented a compelling biologic candidate gene for pharmacogenetic responsiveness to glucocorticoid therapy for asthma. To assess whether common IPO13 DNA sequence variants influence treatment response to inhaled corticosteroids, we genotyped 10 common IPO13 variants in a cohort of children with asthma participating in a clinical trial evaluating the long-term efficacy of inhaled anti-inflammatory medication (including budesonide, a commonly prescribed inhaled GC)[17]. Herein we report that IPO13 variants differentially influenced airway hyperresponsiveness by treatment group, with improvements in AHR noted among subjects who were randomized to either placebo or nedocromil to levels similar to subjects who were randomized to budesonide, suggesting that common IPO13 variants may increase the nuclear bioavailability of endogenous GCs.

## Methods

### Population

CAMP is a multicenter, randomized, double-blinded clinical trial testing the safety and efficacy of inhaled budesonide (200 ug twice daily) vs. nedocromil (8 mg twice daily) vs. placebo over a mean of 4.3 years. Trial design and methodology have been published[17,18]. CAMP enrolled 1,041 children ages 5 to 12 years with mild to moderate asthma. Entry criteria included asthma symptoms and/or medication use for ≥ 6 months in the previous year and airway responsiveness with PC_20 _≤ 12.5 mg/ml. Follow-up visits with spirometry occurred at two and four months and every four months thereafter. Methacholine studies were performed during the run-in period, at 8 months post-randomization, then yearly thereafter. 968 children and 1,518 parents contributed DNA samples, including those of self-reported white (654 children and 950 parents), African-American (131 and 128), and Hispanic (86 and 94) ancestry[19]. Given the relatively small sample sizes of the non-white ethnic groups in CAMP and to avoid spurious association due to population stratification, association analyses were restricted to white probands (Table 1).

**Table 1:** Baseline characteristics of probands in CAMP

**Variables**	**Values**
Sex – N (%)	
Male	393 (60.1%)
Female	261 (39.9%)

Age – Years, Mean (std)	8.9 (2.1)

Age asthma onset – Years, Mean (std)	3.0 (2.4)

Forced expiratory volume (1 sec), post-bronchodilator	
Liters, Mean (std)	1.83 (0.50)
% predicted, Mean (std)	103.5 (12.6)

Methacholine PC_20 _– mg/dl, geometric mean (IQR)	1.08 (0.47 – 2.72)

Total serum IgE levels – IU/L, geometric mean (IQR)	402 (156 – 1069)

std = standard deviation

IQR = interquartile range

### Human Subjects

The Institutional Review Boards of the Brigham and Women's Hospital and of the other CAMP study centers approved this study. Informed assent and consent were obtained from the study participants and their parents to collect DNA for genetic studies.

### SNP genotyping

SNPs were genotyped using SEQUENOM^® ^(Sequenom, San Diego, CA). Primers and reaction conditions are available upon request. One SNP (rs2301993) that failed was genotyped using a TaqMAN™ assay (PE Biosystems, Foster City, CA). All SNP passed quality control, including high genotype completion rates (>95%), less than 1% genotype discordance upon repeat genotyping of a random sample of ~5–10% of the cohort, and lack of parental-offspring genotype incompatibilities.

### SNP discovery

Bidirectional dye-terminator sequencing was performed according to protocol (Applied Biosystems, Foster City, CA) targeting all exons, intron-exon boundaries and 1 kb of flanking genomic sequence at the IPO13 locus in 23 white CAMP subjects selected to ensure representation of all four IPO13 haplotypes. Primers were designed using Primer3, and sequence analysis was performed using the 3130 DNA Analyzer (ABI).

### Statistical analysis

Linkage disequilibrium (LD) and haplotype block analysis was performed using Haploview[20]. We assessed methacholine PC_20 _as the primary outcome of interest as it was the lung-function related phenotype most impacted by inhaled glucocorticoid treatment in the CAMP trial. Methacholine-PC_20 _– the dose of methacholine at which a 20% drop in the Forced Expiratory Volume in one second (FEV_1_) from baseline was observed – was log_10 _transformed to achieve a normal distribution. We adjusted all association tests for baseline (pre-randomization) PC_20_, age, gender, height, study center and visit. The primary clinical trial demonstrated equivalence of nedocromil and placebo with respect changes in airways responsiveness, lung function, and other clinical outcomes[17]. We therefore grouped subjects in these two groups for comparison with those participants randomized to budesonide in order to maximize statistical power. The primary analysis was a longitudinal analysis of methacholine-PC_20 _using the PROC MIXED procedure in SAS assuming a power spatial variance-covariance structure, with random slopes and intercepts estimated using Maximum Likelihood[21,22]. We confirmed results using family-based methods (FBAT-PC[23] in PBAT[24]) to exclude spurious association due to occult population stratification and to perform haplotype association testing. Primary hypothesis testing was performed assuming additive genetic effects. However, because of the relatively small numbers of rare homozygotes within treatment strata, subsequent estimates of genetic effect, tests for gene-by-treatment group interaction, and haplotype analysis were performed assuming a dominant genetic model. Point estimates of the effect of IPO13 polymorphism carrier status on methacholine PC_20 _were determined using generalized linear models (PROC GLM).

To control type I error, the primary analysis of association of IPO13 SNPs with PC_20 _was adjusted for multiple comparisons based on the methods of Nyholt[25] as modified by Li and Ji[26], as implemented in SNPSpD . We first calculated the effective number of independent marker loci tested (M_effLi_), defined by (i) calculating the correlation matrix (i.e. pairwise LD) across all markers using all available genotype data; (ii) measuring the collective correlation across the set of markers as the variance of the eigenvalues from this LD matrix; and (iii) using this measure of collective correlation to calculate the proportional reduction in the number of independent markers. In the CAMP population, the calculated collective correlation was high (variance of the observed eigenvalues = 2.4144) resulting in an estimated M_effLi _of 6 markers, and an adjusted alpha of 0.0085 (see Table 2). We note that because our primary hypothesis testing evaluated two treatment states (budesonide group vs. other), we further adjusted for two sets of tests, resulting in an experiment-wise significance threshold of 0.00425.

**Table 2:** IPO13 SNP pairwise LD correlation matrix used for MeffLi estimation

	1	2	3	4	5	6	7	8	9	10
1	1	-0.49	-0.23	0.49	0.19	0.37	0.54	0.40	0.53	0.39
2	-0.49	1	-0.69	-0.37	0.17	-0.12	-0.33	-0.23	-0.29	-0.16
3	-0.23	-0.69	1	-0.06	-0.12	-0.19	-0.12	-0.31	-0.37	-0.25
4	0.49	-0.37	-0.06	1	0.27	0.54	0.81	0.47	0.64	0.53
5	0.19	0.17	-0.12	0.27	1	0.50	0.28	0.32	0.41	0.41
6	0.37	-0.12	-0.19	0.54	0.50	1	0.56	0.85	0.69	0.89
7	0.54	-0.33	-0.12	0.81	0.28	0.56	1	0.58	0.76	0.56
8	0.40	-0.23	-0.31	0.47	0.32	0.85	0.58	1	0.72	0.86
9	0.53	-0.29	-0.37	0.64	0.41	0.69	0.76	0.72	1	0.74
10	0.39	-0.16	-0.25	0.53	0.41	0.89	0.56	0.86	0.74	1

Matrix derived using all genotyped subjects. Calculated Eigenvalues are 5.2038, 1.5631, 1.1038, 0.7787, 0.5207, 0.3532, 0.1898, 0.1183, 0.0993 and 0.0693, with a resultant observed eigenvalue variance of 2.4231.

## Results

### SNP genotyping

**Figure 1:**
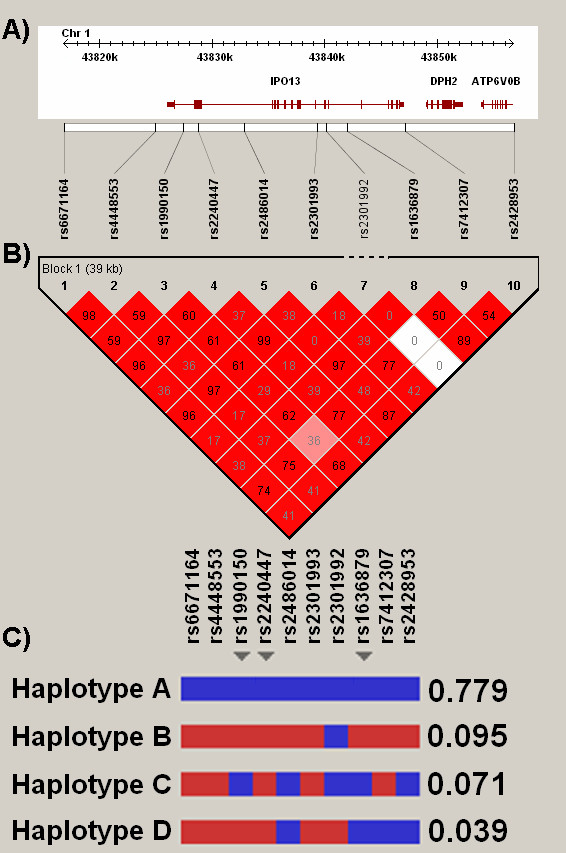
**Genetic structure of IPO13**. Panel A: Relative position of 10 variants genotyped on physical map of chromosome 1p34 region including IPO13. DPH2 = diphthamide biosynthesis protein 2. ATP6V0B = B component of vacuolar ATPase. Panel B: Pair-wise linkage disequilibrium. Numbers denote pair-wise r^2 ^values. Color key denotes strength of pair-wise D'. SNP form one large haplotype block spanning genomic segment. Panel C: IPO13 haplotype structure. Blue = common allele, red = minor allele. Haplotype frequency in CAMP probands of self-reported white ancestry presented on right. Arrowheads denote one of several haplotype-tagging SNP combinations. This 3-SNP combination (rs6671164 – rs199150 – rs2428953) was used for haplotype association analysis in this study.

The ten IPO13 SNPs evaluated in this study were selected from build 119 of dbSNP in order to achieve an average spacing of approximately 1 SNP every 5 kb across the IPO13 locus and its 20 kb flanking sequence (Figure 1a). Among non-Hispanic whites, all 10 SNPs had a minor allele frequency (MAF) of at least 0.05, and pair-wise LD high (median pair-wise D' 0.99, interquartile range of 0.98–1.0; see Figure 1b), suggesting very limited haplotype diversity. Indeed, the 10 SNP form only four common haplotypes (Figure 1c), which can be unambiguously classified by several possible combinations of three tagging-SNPs (one representative set denoted with arrowheads in Figure 1c). The genotyped SNP capture nearly all reported common variation at the IPO13 locus, in that they efficiently tag (r^2 ^> 0.80) all but two of the 21 SNPs with available genotype information in the HapMap families of northern and western European ancestry. This high degree of LD implies substantial redundancy with regard to the number of independent observations made in the association studies to be described below. Using the spectral decomposition of matrices (see Methods and references [25] and [26]) we estimated that the 10 genotyped variants effectively represent only 6 independent markers for association testing.

### Associations of IPO13 with airways hyperresponsiveness in CAMP

As outlined in the introduction, we initially hypothesized that IPO13 polymorphisms would impact individual responsiveness to inhaled corticosteroid responsiveness for the treatment of asthma given the documented role of IPO13 as an active nuclear transporter for receptor-bound glucocorticoid. The CAMP clinical trial demonstrated that inhaled corticosteroid therapy (budesonide 200 ug administered twice daily), as compared to placebo or nedocromil, most significantly impacted methacholine PC_20 _measurements rather than other spirometric measures of lung function[17]. We therefore focused our analysis on whether IPO13 variants impacted PC_20 _over the course of the clinical trial, and whether any observed effects were limited to subjects randomized to inhaled budesonide. As demonstrated in Table 3, all IPO13 variants tested (with the exception of rs2240447) demonstrated some evidence of association with methacholine PC_20 _during the clinical trial, with two variants (rs6671164 and rs2301993) demonstrating significant association after multiple comparison adjustment for 12 tests (6 effective independent markers by 2 treatment strata, corrected alpha = 0.00425). However, in contrast to our assumptions that significant differences would be due to an effect in the budesonide treated group, stratified analysis clearly demonstrated that all of the observed effects were due to differences in the subjects who were not randomized to budesonide. These associations were not likely due to occult population stratification, as follow-up family-based association testing (which is immune to the effects of population stratification) demonstrated similar patterns of association (Table 4).

**Table 3:** Impact of IPO13 polymorphisms on methacholine hyperresponsiveness (PC_20_) in childhood asthma

				Treatment-stratified analysis	
				
SNP	Genic location	MAF	All subjects (n = 654)	Budesonide (n = 214)	Placebo or Nedocramil (n = 440)
rs6671164	5' genomic	0.223	0.002	-	0.004

rs4448553	5' genomic	0.223	0.02	-	0.01

rs1990150	Intron 1	0.148	0.02	-	0.04

rs2240447	Exon 2	0.224	-	-	-

rs2486014	Intron 2	0.097	0.04	-	-

rs2301993	Intron 12	0.222	0.002	-	0.001

rs2301992	Intron 13	0.049	0.02	-	0.03

rs1636879	Intron 14	0.102	0.02	-	0.04

rs7412307	3' genomic	0.184	0.02	-	0.04

rs2428953	3' genomic	0.11	0.002	-	0.005

Results based on repeated-measures analysis assuming an additive genetic model with log_10_-PC_20_. All analyses are adjusted for PC20 prior to treatment randomization, age, gender, height, study center and study visit. All-subject analysis adjusted for treatment-arm assignment (budesonide vs. other). MAF = minor allele frequency. "-" denotes nominal p-value > 0.05.

**Table 4:** Family-based association analysis of IPO13 polymorphisms on methacholine hyperresponsiveness (PC_20_) in childhood asthma.

	Nedocromil/Placebo		Budesonide	
Marker	Number of informative Families	P-value	Number of informative families	P-value

rs6671164	48	0.042	16	0.504

rs4448553	46	0.005	18	0.694

rs1990150	21	0.094	7	0.225

rs2240447	46	0.008	18	0.498

rs2486014	10	0.144	2	0.419

rs2301993	47	0.004	16	0.532

rs2301992	5	0.313	2	0.621

rs1636879	10	0.329	3	0.322

rs7412307	38	0.108	14	0.865

rs2428953	13	0.708	5	0.221

Treatment-stratified analysis of repeated measures of methacholine PC_20 _performed using FBAT-PC as implemented in PBAT.

Though this longitudinal analysis of quantitative measures collected over the course of the clinical trial provides greatest statistical power, clinical interpretation is often more easily appreciated from cross-sectional analyses at discrete time points. As such, we next quantified the impact of IPO13 variants on methacholine PC_20 _in each treatment strata by estimating the SNP-specific fold-change in geometric mean methacholine PC_20 _at 8-months post-randomization (the time point during the clinical trial when maximal treatment response was noted and with fewest missing measurements). As shown in Table 5 and in keeping with the repeated measures analysis described above, carriers of IPO13 variants who were randomized to placebo or nedocromil demonstrated between a 1.6 and 2.3 fold increase in methacholine PC_20 _compared to non-carriers for most SNP, whereas no significant difference in methacholine PC_20 _was noted for any SNP among subjects randomized to budesonide. Subtle differences in statistical significance (but not genetic effect estimates) were observed between the repeated measures and 8-month analyses (for example rs2301992 was not statistically significant in the 8-month cross-section analysis, while rs2240447 and rs2486014 demonstrated statistical significance only in the repeated measures analysis), suggesting instability in the variances in effect for these SNP in comparison to the others. Nonetheless, the general patterns across the locus were similar in both analyses and suggest that common IPO13 variants are associated with reduced airways responsiveness.

**Table 5:** Fold-change in methacholine PC_20 _by treatment group at 8-months post-randomization among IPO13 variant carriers

	Budesonide (n = 214)		Nedocromil/Placebo (n = 440)		
SNP	Fold change (95% CI)	p-value	Fold change (95% CI)	p-value	Test for interaction
rs6671164	0.81 (0.38 – 1.68)	0.56	1.65 (1.05 – 2.61)	0.03	0.13

rs4448553	0.84 (0.40 – 1.77)	0.65	1.58 (1.00 – 2.51)	0.05	0.20

rs1990150	0.72 (0.31 – 1.69)	0.45	2.25 (1.35 – 3.74)	0.002	0.02

rs2240447	0.72 (0.34 – 1.53)	0.39	1.82 (1.14 – 2.89)	0.01	0.05

rs2486014	0.99 (0.37 – 2.62)	0.98	1.97 (1.10 – 3.53)	0.02	0.21

rs2301993	0.83 (0.39 – 1.79)	0.64	1.80 (1.13 – 2.86)	0.01	0.11

rs2301992	0.48 (0.15 – 1.52)	0.21	1.64 (0.78 – 3.47)	0.19	0.08

rs1636879	1.02 (0.38 – 2.74)	0.96	2.14 (1.20 – 3.83)	0.01	0.11

rs7412307	0.74 (0.34 – 1.60)	0.44	1.84 (1.14 – 2.96)	0.01	0.06

rs2428953	0.67 (0.26 – 1.70)	0.40	2.30 (1.32 – 4.00)	0.003	0.02

Models adjusted for baseline log10-PC_20_, age, gender, height and clinic. Fold-change and confidence limits derived using beta-estimates derived from generalized linear models.

**Figure 2:**
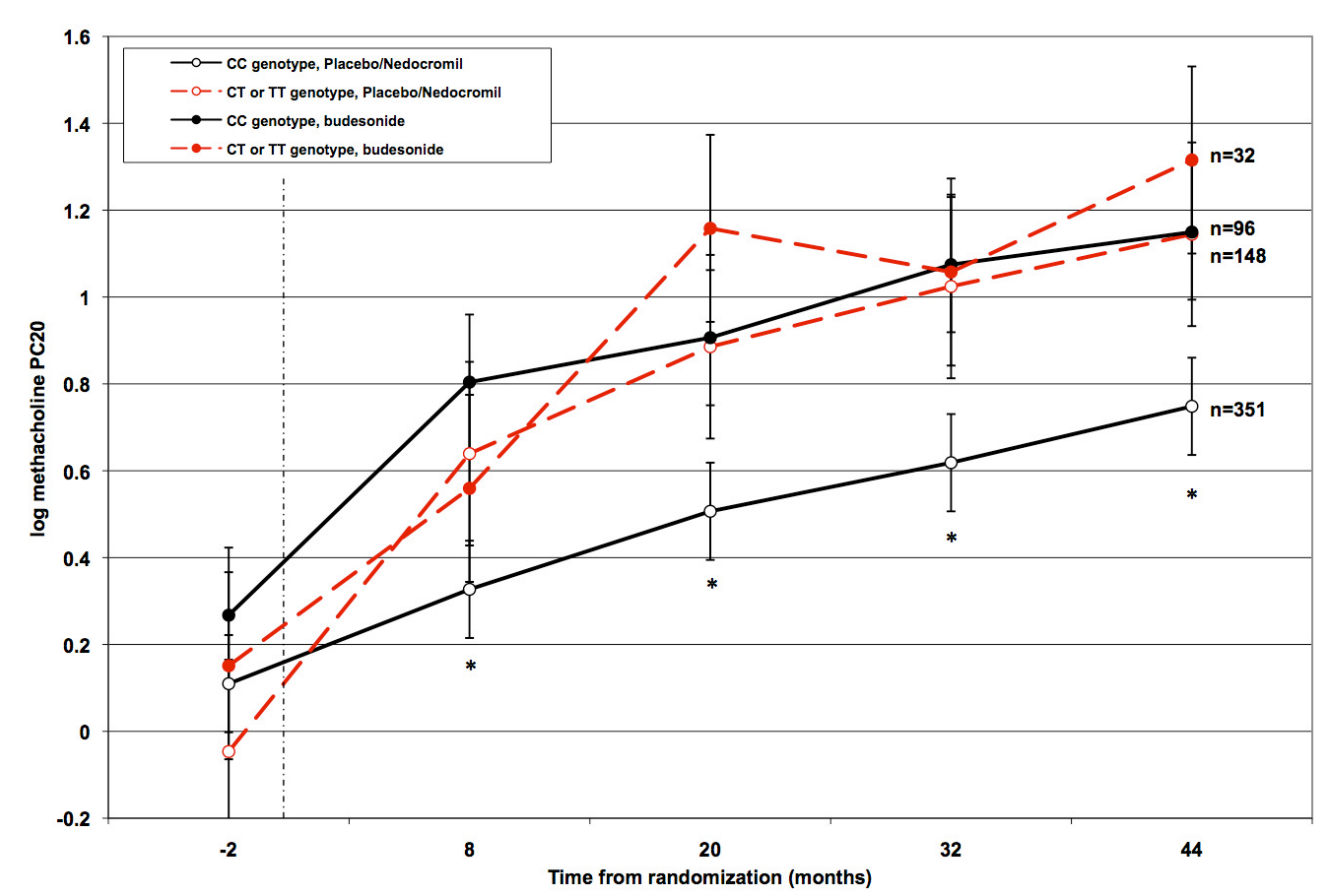
**Impact of IPO13 polymorphism rs2428953 on airway hyperresponsiveness**. Mean log(methacholine PC_20_) values and SEM. Common genotype (CC) denoted by solid black lines, heterozygotes and TT homozygotes denoted by dashed red lines. Open circles denote placebo/nedocromil groups, closed circles represent budesonide groups. Vertical line at 0 months denotes time of randomization. Airway hyperresponsiveness was significantly different (p < 0.05) between the placebo/nedocromil subjects with CC genotype and all other subjects at all time points following randomization, as denoted by (*).

Figure 2 illustrates the differential effects of one representative polymorphism (rs2428953) on airway responsiveness over the course of the clinical trial. As can be seen, T-allele carriers on placebo or nedocromil had improvements in PC_20 _values over the course of the trial that approached those observed among subjects who were treated with budesonide. In keeping with the longitudinal analysis, similar profiles were observed for the other IPO13 SNP, with carriers of the minor alleles at all loci (with the exception of rs2240447 and rs2301993) randomized to placebo or nedocromil demonstrating significantly less airways responsiveness compared to non-carriers (data not shown). From these data, we conclude that IPO13 variation influences the natural progression of methacholine PC_20 _among children with asthma, resulting in significantly less severe airway hyperresponsiveness, and approaching levels achieved with inhaled corticosteroid therapy. These data also suggest that the addition of inhaled corticosteroids confers no additional benefit among IPO13 carriers, though our study is underpowered to formally test that specific interaction. We also note that airway hyperresponsiveness was lowest among subjects homozygous for IPO13 variants (for example, the mean logPC_20 _was 1.31 among SNP rs2428953 TT homozygotes compared to 0.77 among heterozygotes), suggesting an additive genetic relationship, though the number of subjects with rare homozygous genotypes was generally too small (13 in the non-budesonide group, 2 in the budesonide group) to make formal statements regarding the significance of this observation.

### Haplotype association analysis

As described above, four common (frequency > 1%) IPO13 haplotypes are observed in this cohort. We assessed whether the associations observed above could be attributed to specific haplotypes, we preformed a family-based haplotype association test using one representative set of haplotype-tagging SNP (rs1990150, rs2240447, and rs1636879 – arrow heads in Figure 1c). Similar to the single SNP analysis, treatment-stratified haplotype analysis demonstrated association with methacholine PC_20 _among subjects randomized to placebo or nedocromil only (global p-value for test of association of IPO13 locus = 0.02), but not among subjects randomized to budesonide (p = 0.58). Inspection of the haplotype-specific p-values (Table 6) suggests that the primary association is with haplotype B (haplotype specific p-value = 0.02), though the relatively small number of informative families available for evaluating haplotypes C and D preclude definitive statements regarding these later haplotypes.

**Table 6:** Family-based haplotype association analysis of IPO13 polymorphisms on methacholine hyperresponsiveness (PC20) in childhood asthma.

	Nedocromil/Placebo (global p-value = 0.02)		Budesonide (global p-value = 0.58)	
Haplotype	Number of informative families	P-value	Number of informative families	P-value

A	136	0.41	53	0.33

B	84	0.02	33	0.53

C	69	0.17	20	0.41

D	43	0.48	18	0.50

Haplotype analysis was performed using one of several haplotype-tagging SNP combinations: rs6671164 – rs199150 – rs2428953. The haplotype designations A through D correspond to those illustrated in Figure 1.

### SNP discovery

Resequencing of the IPO13 locus in 23 CAMP subjects selected to ensure representation of all four common IPO13 haplotypes identified 8 variants that had not been genotyped, including four not present in the dbSNP database (Table 7). Two were potentially functional (a non-synonymous Asp891Ser substitution and a highly conserved non-coding variant), though their low frequency (each was observed only once) and presence only on the common haplotype A background (not the PC_20 _associated background) suggest that they are not responsible for the observed associations with PC_20_.

**Table 7:** IPO13 polymorphisms identified through SNP discovery effort

SNP	Alleles	MAF	rs number	Chr 1 position (in bp from pter)	Conserved base*
-1022 C>G	C/G	0.043	-	44184837	No

Asp415Asp	C/T	0.043	rs17402858	44195209	Yes

IVS11-13 C>T	C/T	0.050	-	44196992	No

IVS14+14 A>G	A/G	0.043	rs2906596	44199535	No

IVS17+46 G>C	G/C	0.174	rs4660759	44205087	No

Asp891Ser	G/T	0.022	-	44205631	Yes

g.20431 A>G	A/G	0.022	-	44206290	Yes

g.21341 A>G	A/G	0.087	rs2486007	44207200	No

The nomenclature for discovered variation provided here follows that recommended by den Dunnen and Antonarakis[29]. Asp = Aspartic Acid; Ser = Serine; IVS denotes intervening sequence (i.e. intronic) SNP. MAF = minor allele frequency.

* Conservation based on 8-species sequence alignment as derived using Phylo-HMMs[30].

## Discussion

Herein, we observed significant associations of common IPO13 polymorphisms with airway responsiveness (the most dynamic treatment response phenotype in the CAMP clinical trial) among children with mild-to-moderate asthma. These associations were observed exclusively among those children who were not randomized to inhaled GCs, and persisted over the ~4.5 years of clinical observation. The genetic effects conferred by these IPO13 variants were clinically significant, with an average 1.5–2.1 fold increase in mean PC_20 _among carriers of IPO13 variants.

IPO13 has been functionally characterized as a primary regulator of GC-bound GR transport across the nuclear membrane. Inhibition of lung epithelial cell IPO13 production inhibits nuclear translocation of GR from the cytoplasm and subsequent GC-mediated silencing of inflammatory cytokine production[13], suggesting that the normal anti-inflammatory response induced by GC is dependent on normal IPO13 function. In light of these observations, it is curious that the genetic effects observed in the current study were observed only among subjects who were not taking daily corticosteroids. We propose two possible mechanisms to explain these findings. The first is developmental. IPO13 was first identified in studies of lung development, where IPO13 (initially known as LGL2) was found to be differentially expressed in fetal rat lung cell culture[12]. It is conceivable that IPO13 variation impacts airway hyperresponsiveness by altering airway anatomy through changes during airway morphogenesis and development. Though plausible, the absence of association of IPO13 variants with baseline lung function in the current study (data not shown) suggests that this is not likely the case. However, given the diverse roles of glucocorticoid during lung morphogenesis (see Introduction) and the considerable impact on lung development by aberrant perinatal glucocorticoid exposure[27,28], we are hesitant to completely discount this possibility at this time.

A second possible mechanism of action is that IPO13 variants improve airway hyperresponsiveness by enhancing the local anti-inflammatory effects of circulating, endogenous GCs by facilitating increased GR transport into the nucleus and thus increasing the effective bioavailability of endogenous GC. Supporting this hypothesis is the observation that carriers of IPO13 variants who were not on inhaled steroid exhibited improvements in airway responsiveness over the course of the clinical trial that approached those for subjects who were taking inhaled steroids (see Figure 2), suggesting that that IPO13 variation enhances endogenous GC nuclear availability to therapeutic levels. It has been previously demonstrated that nucleocytoplasmic shuttling of IPO13 is developmentally regulated and highly variable in rat lung[12,13]. Sequence variation could potentially influence this regulation by increasing nuclear membrane availability through increased IPO13 expression or by altering the kinetic properties of GR transport by influencing GR binding affinities. We note that the paucity of coding variation at the IPO13 identified through our resequencing efforts suggests that the genetic effects observed are likely due to regulatory variation rather than structural changes. Though studies are currently ongoing to explore these possibilities, we note that because none of the airway responsiveness-associated non-coding variants map to highly conserved genetic sequence and are not predicted to harbor transcription factor binding sites, it is unlikely that we have as of yet identified a putative functional variant.

It is noteworthy that the associated haplotype block not only spans the IPO13 locus, but two neighboring genes as well: ATP6V0B and DHP2 (Figure 1). Though it is not possible to completely exclude these other genes as functionally responsible for the observed associations, it is unlikely to be the case. The motivation for studying these polymorphisms was the recognition of IPO13 as the primary nuclear transporter for steroid-bound glucocorticoid. Had we identified these variants through a hypothesis-free approach (i.e. a genome-wide study), the pretest probability for each gene in the region would be similar. However, because these SNPs were chosen due to the biologic prior on IPO13, it would be improbable that the true functional effects would be mediated through a neighboring gene. Unlike IPO13, there is little biological evidence to support either ATP6V0B or DHP2 in either the pathogenesis of airways responsiveness or glucocorticoid pharmacogenetics. ATP6V0B is a subunit of the vacuolar-type H(+)-ATPase (V-ATPase) multisubunit enzyme, responsible for organelle acidification. DHP2 encodes a protein involved in diphthamide biosynthesis that confers resistance in yeast to the effects of diphtheria toxin. Though surveys of genomic databases (including UniGene and GEO) suggest that ATP6V06 is ubiquitously expressed and DHP2 is weakly expressed in the lung, there is little reason to suspect either as the responsible locus.

In genetic association studies of complex traits such as airway hyperresponsiveness and pharmacogenetic responses, it is important to consider a variety of methodological and statistical issues that can hamper proper interpretation of observed findings. Two features of the current study warrant particular attention: phenotype misclassification and statistical power. Most observational studies of the genetics of asthma attempt to avoid confounding of lung phenotype measurements by medication use by performing spirometric and airway hyperresponsiveness measurements following a short period (less than 24–48 hours) off anti-asthma controller medications. However, due to safety consideration, long-term avoidance of asthma controller medication is typically not permitted. The randomized, placebo-controlled trial is perhaps the only setting in which such confounding can be eliminated, and is a major strength of the study presented here. It is directly a result of the availability of ~4.5 years of repeated methacholine challenge measurements off anti-inflammatory agents among more than two-thirds of CAMP participants who were randomized to placebo or nedocromil that enabled detection of the observed associations in this study. In light of the differences in genetic effect observed across treatment arms, we stress that future attempts to replicate our findings should use approaches that address this issues, as replication may only be possible when proper adjustment for glucocorticoid use are made.

Subjects randomized to inhaled budesonide represent only 1/3 of the cohort. We recognize that this relatively smaller sample size is inadequately powered to detect strong genetic effects with alleles of modest frequency, and that it is possible that an effect similar to that observed among those not on steroids could potentially have been observed among steroid users had the number of subjects in this latter group approached those of the former. However, we note that sample size impacts only the ability to claim a statistically significant difference in genotype effect but does not in any way influence the absolute effect observed. In this study, the trends of association among those on budesonide were quite dissimilar to those observed among subjects not on budesonide, with all variants actually conferring greater airway responsiveness (though the confidence intervals in this group are quite broad and all span the null). Therefore, while we stress that our conclusions reported herein apply only to those subjects in the non-budesonide arm, it is likely that differential effects of these alleles are present between subjects who were and were not taking steroids.

## Conclusion

We have demonstrated that genetic variation in IPO13 is associated with reduced airway hyperresponsiveness among children with mild-to-moderate asthma who are not using long-term inhaled corticosteroids. The degree of this reduction is similar to the improvements noted among those children on long-term corticosteroids, supporting the notion that IPO13 variation improves endogenous glucocorticoid bioavailability in the cell nucleus. We are currently performing functional studies using patient-derived cell lines representing the spectrum of IPO13 genetic variation to explore this possibility.

## Abbreviations

AHR: Airway hyperresponsiveness; CAMP: Childhood Asthma Management Program; GC: Glucocorticoid; GR: glucocorticoid receptor; IPO13: Importin-13; Kb: kilobase; LD: linkage disequilibrium; MAF: minor allele frequency; PC_20_: dose of methacholine bonchoprovocation test resulting in 20% in baseline forced expiratory volume in one second; SNP: single nucleotide polymorphism.

## Competing interests

The authors declare that they have no competing interests.

## Authors' contributions

BAR designed and supervised the genotyping and sequencing studies, performed the primary data analysis and drafted the manuscript. KVS and JLS performed the statistical analysis. BAR, KT, FK and STW conceived of the study, participated in its design and coordination. All authors read and approved the final manuscript.
